# G3BP1 regulates breast cancer cell proliferation and metastasis by modulating PKCζ

**DOI:** 10.3389/fgene.2022.1034889

**Published:** 2022-10-18

**Authors:** Shuang Liu, Shaoping Tian, Tianyu Lin, Xin He, Justin Eze Ideozu, Rui Wang, Yong Wang, Dan Yue, Hua Geng

**Affiliations:** ^1^ Department of Microbiology, School of Medical Laboratory, Tianjin Medical University, Tianjin, China; ^2^ Department of Pediatrics, School of Medicine and Public Health, University of Wisconsin-Madison, Madison, WI, United States; ^3^ Genomic Medicine, Genomic Research Center, AbbVie, North Chicago, IL, United States; ^4^ Department of Urology, The Second Hospital of Tianjin Medical University, Tianjin Medical University, Tianjin, China; ^5^ Center for Intestinal and Liver Inflammation Research, Stanley Manne Children’s Research Institute, Ann & Robert H. Lurie Children’s Hospital of Chicago, Chicago, IL, United States; ^6^ Center Department of Pediatrics, Feinberg School of Medicine, Northwestern University, Chicago, IL, United States

**Keywords:** G3BP1, PKCζ, breast cancer, metastasis, proliferation

## Abstract

Breast cancer is a leading cause of death and morbidity among female cancers. Several factors, including hormone levels, lifestyle, and dysregulated RNA-binding proteins, have been associated with the development of breast cancer. Ras-GTPase-activating protein SH3 domain-binding protein 1 (G3BP1) and protein kinase C, Zeta isoform (PKCζ) are oncogenes implicated in numerous cancers, including breast cancer. However, their interaction and role in promoting breast cancer proliferation and metastasis have not been well-characterized. In the present study, we demonstrated that G3BP1 expression was elevated in breast cancer and that knockdown of G3BP1 diminished the proliferation and metastasis of breast cancer cells. Mechanistically, we identified proliferation and a series of metastasis-related properties, including chemotaxis, migration, Golgi polarity localization, and actin polymerization, that were modulated by G3BP1 knockdown. We found that G3BP1 and PKCζ were co-localized and interacted intracellularly, and they co-underwent membrane translocation under EGF stimulation. Following the knockdown of G3BP1, we observed the membrane translocation and phosphorylation of PKCζ were significantly impaired, suggesting that G3BP1 regulates the activation of PKCζ. Our findings indicate that G3BP1 plays multiple roles in breast cancer cell proliferation and metastasis. The activation of PKCζ by G3BP1 may be the specific mechanism underlying the process.

## Introduction

Breast cancer is the most diagnosed cancer in the world and the leading cause of morbidity and mortality among female cancers, according to the 2020 global cancer data statistics (Sung et al., 2021). The development of breast cancer is associated with a variety of factors including hormone levels, lifestyle, and genetic mutations (Metcalfe et al., 2010; Bray et al., 2021). Surgery and chemotherapy are the main treatments for early-stage breast cancer, while patients with advanced metastatic breast cancer receive radiotherapy and chemotherapy (Miller et al., 2019). Although remarkable progress has been made towards advancing treatment for breast cancer, variability in responses to chemotherapy and chemotherapy resistance among patients are major challenges (An et al., 2021).

Ras-GTPase-activating protein SH3 domain-binding protein 1 (G3BP1) is the first identified Ras GAP SH3 structural domain-specific binding protein that is involved in the downstream of the Ras signaling pathway (Irvine et al., 2004). Ras is an important member of the small G family with GTPase activity and is involved in regulating various cellular functions related to cell growth, differentiation, apoptosis, and cytoskeletal remodeling through the activation of signal transduction cascade reactions by receptor tyrosine kinases (Bos et al., 2007). G3BP1 plays a central role in the regulation of RNA stability and the formation of stress granules. Beyond the multifunctional role of G3BP1, its overexpression has been observed in several tumors (Guitard et al., 2001). This suggests that G3BP1 may be an important regulatory molecule in tumorigenesis and progression. However, the specific mechanism by which G3BP1 plays a role in breast cancer cell proliferation and metastasis remains unclear.

GTP-bound Ras has been reported to exert its cellular functions by binding to its effectors such as protein kinase C, Zeta isoform (PKCζ) (Rajalingam et al., 2007). PKCζ is an atypical isoform of the protein kinase C superfamily that plays multiple roles in physiological processes and diseases. Studies have shown that PKCζ is a key molecule in insulin-mediated glucose uptake (Liu et al., 2010). It plays a role in glucose metabolism by regulating glucose uptake proteins (Manna and Jain, 2013). In endothelial cells, PKCζ can promote angiopoietin-1 (Ang-1)-induced angiogenesis (Oubaha et al., 2012). PKCζ may play a unique function in atherosclerosis (Abe and Berk, 2014). Meanwhile, PKCζ has attracted more attention in tumor research, it has been reported that PKC may be a key molecule in the regulation of melanoma proliferation by C2 ceramide (Ghosh et al., 2020). Knockdown of PKCζ impaired the growth and lymphatic metastasis of prostate cancer in xenograft models (Zang et al., 2019). PKCζ is capable of participating in the metabolic reprogramming of glutamine in tumors (Ma et al., 2013) and regulates fatty acid β-oxidation through phosphorylation of SITR6 (Gao et al., 2019). In addition, PKCζ expression is significantly higher in leukemia patients and associated with increased mercaptopurine sensitivity (Hartsink-Segers et al., 2015).

In this study, we demonstrated that G3BP1 was a key molecule regulating breast cancer cell proliferation and metastasis and that its role in breast cancer cells may be exerted by activating PKCζ.

## Materials and methods

### Cell culture and establishment of stable cell lines

Human breast cancer cell line MDA-MB-231 and human embryonic kidney cell HEK-293T were obtained from American Type Culture Collection (ATCC, United States). The cells were grown in Dulbecco’s Modified Eagle’s Medium (DMEM) (Biological Industries, Israel) supplemented with 10% fetal bovine serum (Biological Industries, Israel) and 1% penicillin/streptomycin (Biological Industries, Israel) in 37°C incubator with 5% CO_2._ To generate stable cell lines, lentiviral packaging plasmids (psPAX2 and pMD2.G) were transfected with the target plasmids using Lipofectamine 2000(Invitrogen, United States) in 293T cells. Lentiviral supernatants were collected, centrifuged, and filtered through a 0.45 μm filter. MDA-MB-231 cells were then infected with lentiviral supernatant for 24 h and selected with 2 μg/ml puromycin (Sangon Biotech, China) or 1200 μg/ml G418 (MDBio Inc., China) for 14 days, followed by maintenance with 0.5 μg/ml puromycin or 300 μg/ml G418. The shRNA sequences targeting G3BP1 are listed in (Supplementary Table S1).

### Small interfering RNA

Small interfering RNAs targeting human G3BP1 were designed and synthesized by GENECHEM (Shanghai GENECHEM, China). The sequences are listed in (Supplementary Table S2) and an unrelated sequence with the same GC content was used as the negative control (scrambled siRNA). For transfection experiments using siRNA, an appropriate amount of cells were inoculated in 6-well plates, and the convergence rate reached 30–50% after 24 h culture, and the antibiotic-free medium was changed before transfection. Lipofectamine 2000 (Invitrogen, United States) was diluted with opti-MEM (Invitrogen, United States), and incubated at room temperature for 5 min. After that, it was mixed with diluted 100 pmol siRNA and incubated at room temperature for 20 min. The compound was added to the cell culture medium, cultured at a 37°C and 5% CO_2._ Incubator for 4–6 h, and then replaced with an ordinary antibiotic-free culture medium. The cells were transfected for 48 h and directly used in the experiment.

### Western blot

The cells were washed twice in ice-cold PBS and lysed in 1×SDS lysis buffer supplemented with 1× protease inhibitor cocktail (Roche Applied Science, Germany) on ice for 10 min. The mixture was then collected in 1.5 ml Eppendorf tubes, sonicated for 15 s under ice bath conditions, followed by denaturation at 95°C for 10 min. The protein concentration was determined by the BCA method (BCA Protein Assay Kit, Pierce, United States). The protein samples were separated by SDS-PAGE and blotted onto polyvinylidene difluoride membranes (Millipore, United States). Each membrane was sealed with 5% skim milk or BSA at room temperature for 2 h and then incubated overnight with the primary antibody to G3BP1 (Santa Cruz Biotechnology, United States, sc-365338; 1:1000), PKCζ (Abcam, UK, ab108970; 1:3000), p-PKCζ (Abcam, UK, ab62372; 1:3000), GAPDH (Affinity, United States, AF7021; 1:3000). On the second day, after the membranes were washed with TBST to remove the unbound primary antibody, the membranes were incubated at room temperature with conjugated secondary antibody for 1 h, and protein expression was detected using ECL (Affinity Biosciences, United States).

### Immunoprecipitation

The MDA-MB-231 cells were washed 5 times with ice PBS, and 500 μl of pre-cooled Tris-Triton cell lysis buffer (40 mM Tris, 120 mM NaCl, 1% Triton X-100, 1 mM NaF, 1 mM Na_3_VO_4_, 1×cocktail) was added to lysate the cells. After spinning and mixing for 30 min, the sample was centrifuged at 12000g for 15 min and the supernatant was collected. Then the sample was mixed with 5 μg PKCζ antibody (Abcam, UK, ab59364). Homogenous IgG antibody (Cell Signaling Technology, United States, #2729) was then added to the control group and the mixture was incubated at 4°C for 6 h with slow shaking. Then, 50 μl protein A/G magnetic beads (Invitrogen, United States) was added to each group before overnight incubation at 4°C. On the second day, the supernatant was removed from the magnetic rack, and 50 μl of 2× loading buffer was added to the magnetic beads after washing 4 times with cell lysis buffer. The magnetic beads and immune complexes were then incubated at 95°C for 5 min. The western blot analysis was used to detect both the input and immunoprecipitation protein lysis.

### Chemotaxis assay

EGF (Millipore Chemicon, United States) diluted with binding medium (DMEM, 0.1%BSA, 25 mM HEPES) at the concentration of 0 ng/ml, 1 ng/ml, 10 ng/ml, and 100 ng/ml were placed in the lower chamber of the chemotactic chamber (Neuro Probe, China), with binding medium added as the negative control. About 5 ×10^5^/ml MDA-MB- 231 cells were re-suspended with DMEM medium and added into the upper chamber of the chemotactic chamber. Polycarbonate films (Neuro Probe, China) treated overnight with Fibronectin (Sigma, United States) at 4°C were placed between the upper and lower chambers of chemotactic cells. Then the chemotactic chamber was incubated for 3 h in a 37°C incubator with 5% CO_2_. The cells passing through the membrane were fixed and stained, subsequently counted in three fields randomly by microscope.

### Wound healing assay

After digestion with 0.25% trypsin (Biological Industries, Israel), MDA-MB-231 cells at the logarithmic growth stage were re-suspended with DMEM and inoculated into a six-well plate. After transfection with small interfering RNA targeting G3BP1 and control sequence, the cells were scratched evenly in the cell culture dish with a 10 µl tip, and the distance of cell movement was recorded every 3 h. Three random distances were recorded to count the differences between the two groups of cells.

### Cell polarity assay

One day before the experiment, the cells transfected with siG3BP1 for 48 h were placed in a 12-well plate with a small cover glass, and the cells were scratched evenly with a 10 μl tip. After 20 min and 6 h, the cells were fixed with 4% PFA, then 0.2% Triton X-100 was used to permeabilize cells, and the nucleus was stained with DAPI (Solarbio, China). Giantin (Abcam, UK, ab80864; 1:1000) was then added followed by overnight incubation in a 4°C refrigerator. The next day, the tablets were sealed with anti-fluorescence attenuated media and observed with a confocal microscope.

### Actin polymerization assay

MDA-MB-231 cells with an appropriate density were seeded in a cell culture dish and starved in a serum-free medium for 3 h. EGF (50ng/ml) was used to stimulate the cells for 0, 4, 8, 15, 30, 60, 120, and 300 s, before the reaction was terminated by ice-cold PBS. The cells were fixed with 3.7%–4% PFA, and F-buffer (5 mM EGTA, 2 mM MgCl_2_, PBS) containing 0.2% Triton X-100 were used to permeabilize cells. After washing with F-buffer five times, Alexa Flour 568 phalloidin (Invitrogen, United States) was added, followed by 1 h incubation at room temperature with protection from light. The aggregation changes of actin in the cells were observed under a confocal microscope. In addition, the combined phalloidin was extracted with methanol at 4°C for 1 h and then used to detect changes in fluorescence intensity. After the remaining cells were washed with F-buffer, the protein was extracted and the concentration was determined using the BCA method.

### Immunofluorescence

MDA-MB-231 cells were plated on poly-d-lysine-coated coverslips in 12-well culture plates and incubated for 24 h at 37°C. Then, cells were fixed with methanol for 10 min at -20°C, permeabilized with 0.2% Triton X-100 in PBS for 10 min and blocked with 3% BSA in PBS. Following PKCζ antibody (Abcam, UK, ab59364; 1:50) and G3BP1 antibody (Santa Cruz Biotechnology, sc-365338; 1:10) incubation overnight at 4°C, the cells were stained with Alexa-Fluor 488 and 546 conjugated secondary antibodies (Invitrogen, United States) for 1 h at room temperature. Fluorescent signals were captured by a confocal microscope.

### Human patients

Human patient cohorts were used to explore the clinical importance of G3BP1 expression in breast cancer. We collected 68 cases of primary breast cancer tissues and 7 cases of benign breast tissues. All breast cancer tissues were surgically excised and paraffin-embedded at Tianjin Medical University Cancer Hospital, with patient consent and ethics committee approval. The inclusion criteria were: patients with pathological diagnosis of breast cancer and patients who underwent radical mastectomy with no adjuvant treatment before and after surgery. In addition, clinicopathological parameters of the patients were collected, including age, tumor size, tumor stage, and metastasis.

### Immunohistochemistry

Tissue wax blocks were cut to 5 μm thickness, then dewaxed in xylene and hydrated in ethanol at different concentrations. The tissue sections were subsequently immersed in sodium citrate to repair antigens at high temperature, and after cooling to room temperature, the tissues were treated with 3% peroxidase blocking agent and finally blocked with 2% BSA for non-specific antigenic sites. Then the primary antibody against G3BP1 was added to the tissue before overnight incubation at 4°C. On the second day, after washing with PBS three times to remove the primary antibody, the secondary antibody (ZSCB-BIO, China) was added and incubated at 37°C for 20 min. The reaction was visualized by the HRP DAB Detection Kit (ZSGB-BIO, China) and counterstained with hematoxylin. After the sections were sealed with neutral balsam, the histopathological changes of the different tissues were observed under an optical microscope. The results of the staining were scored as: 0 points for unstained, one points for light yellow, two points for yellow or yellowish-brown, and three points for brown. Greater than or equal to one point was judged as positive staining.

### CCK-8 proliferation assay

After starvation with serum-free DMEM medium, MDA-MB-231 cells were grown in 96-well plates, with six replicate wells of each cell type. After the cells were plastered, 10 μl of cell counting kit-8 (Biosharp, China) was added to each 100 μl of medium. Cells were incubated at 37°C for 2 h and the absorbance was measured at 450 nm. All plates were assayed at 0, 24, 48, 72, and 96 h time points to observe cell proliferation.

### Invasion assay

Invasion assay was performed using transwell chambers (Millipore, United States) containing polycarbonate membranes with 8 μm pores. After the appropriate proportion of diluted Matrigel (Becton, Dickinson and Company, United States) was spread in the chambers and placed in a 37°C incubator for complete solidification. The MDA-MB-231 cells were starved in serum-free DMEM medium for 4 h and then added to the upper chamber, while the DMEM medium containing 20% FBS was added to the lower chamber. After incubation for 15 h at 37°C, cells in the upper layer of the chambers were wiped off, while cells attached to the submembrane surface were fixed and stained, and then counted in five randomly selected areas. Each group of cells was repeated in three chambers.

### Xenograft tumor growth

Healthy BALB/c nude mice (6–8 weeks old, female) were purchased from Beijing Vital River Laboratory Animal Technology Co. Ltd. (Beijing, China). Mice were randomly divided into a control group (MDA-MB-231-pLKO.1-luc) and an experimental group (MDA-MB-231-pLKO.1-shG3BP1-luc) (*n* = 8 each group), and were marked. Luciferase-labeled G3BP1 knockdown and control of MDA-MB-231 cells were stably constructed (MDA-MB-231-pLKO.1-shG3BP1-luc and MDA-MB-231-pLKO.1-luc), the 1×10^6^ cells were mixed with Matrigel and injected in mammary fat pads of BALB/c nude mice. Animal experiments complied with the ARRIVE guidelines and were carried out following the National Institutes of Health guide for the care and use of laboratory animals (NIH Publications No. 8023, revised 1978). The experimental units that died during the operation were excluded. After 10 weeks, mice were intraperitoneally injected with luciferin (Promega, United States) and live IVIS imaging system was performed to detect the bioluminescence intensity of the primary tumor (*n* = 5 each group). Tumor tissue was peeled from the mammary glands of two groups of mice for weighing to compare the difference in tumor growth (*n* = 5 each group). The difference between the two groups was analyzed using Student’s *t*-test. Non-results reader was aware of the group allocation at the stages of the outcome assessment and the data analysis. The chosen numbers per group were based on findings in previous studies and a subsequent sample size analysis.

#### *In silico* functional analysis

We explored mRNA and protein expression of G3BP1 in breast cancer and normal tissues using The University of Alabama at Birmingham Cancer data analysis Portal (UALCAN) (http://ualcan.path.uab.edu) (Chandrashekar et al., 2017). This is a comprehensive, user-friendly, and interactive web resource for analyzing cancer OMICS datasets. The KM-plotter tool (https://kmplot.com/analysis/) was utilized to explore the relationship between the expression of G3BP1 and the survival of breast cancer patients. Further, we analyzed the expression of G3BP1 in pan-cancer using the Xiantao web (https://www.xiantao.love) and focused on exploring the expression of G3BP1 in breast cancer versus paired normal samples and the impact of G3BP1 expression on the overall survival of breast cancer patients.

### IP-MS

The exogenous PKCζ was transfected in HEK-293T cells, and the protein lysate was collected after 24 h. Then the PKCζ antibody was added to pull down the PKCζ-bound proteins and gel electrophoresis was performed. After electrophoresis, the gel was stained with Komas Brilliant Blue for 2 h and decolorized with glacial acetic acid for about 1 h. The bands on the gel were visualized, and the interacting proteins of PKCζ were identified by Mass Spectrometry.

## Statistical analysis

Statistical analysis was performed using the SPSS (Version 20) program or GraphPad Prism software (Version 8). The Student’s *t*-test and two-way ANOVA were used to determine statistical significance. Differences of *p* < 0.05 were considered statistically significant.

## Results

### G3BP1 is highly expressed in breast malignancies and correlates with poor survival

**FIGURE 1:**
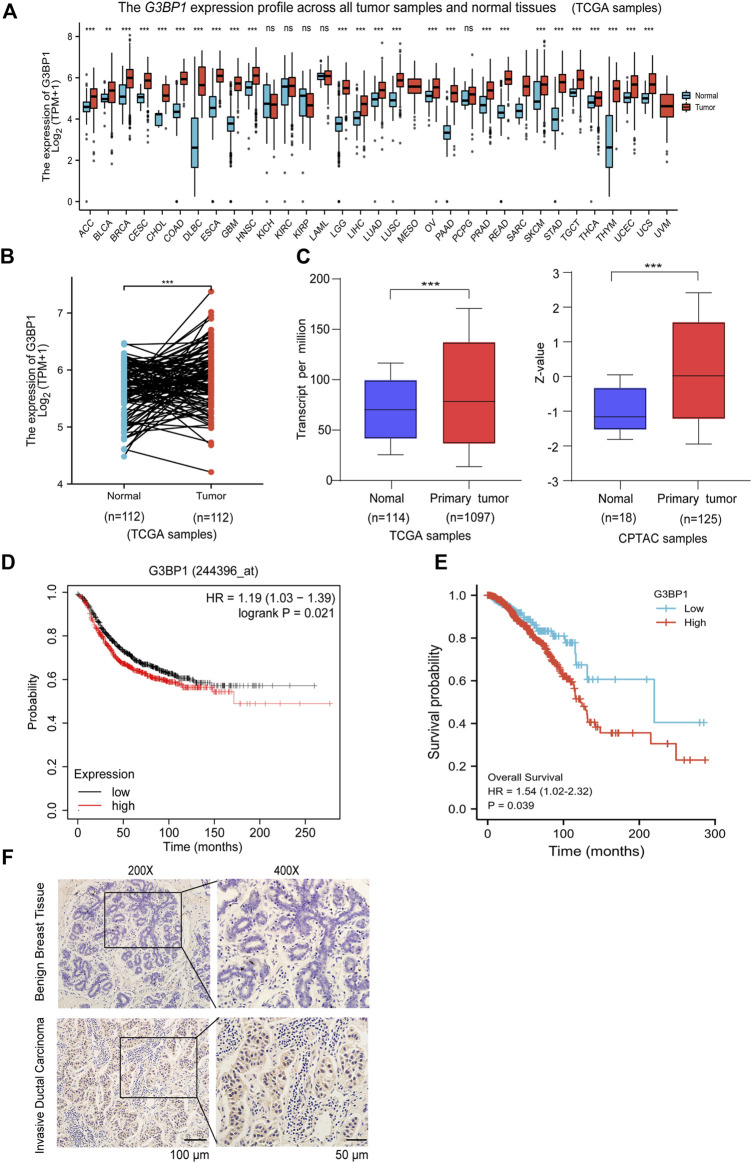
G3BP1 is highly expressed in breast cancer **(A)** Expression levels of G3BP1 mRNA in pan-cancer (https://www.xiantao.love) **, *p* < 0.01, ***, *p* < 0.001, ns: no significance. **(B)** Expression of G3BP1 mRNA in 112 groups of breast cancer and normal paired samples (https://www.xiantao.love) ***, *p* < 0.001. **(C)** Differences in expression of G3BP1 mRNA and protein between normal and primary breast cancer (http://ualcan.path.uab. edu) ***, *p* < 0.001, ***, *p* < 0.001. **(D–E)** Overall survival of high and low G3BP1 expression groups in breast cancer patients (https://kmplot.com/analysis/) (https://www.xiantao.love) *, *p* < 0.05. **(F)** Representative images of G3BP1 immunohistochemical staining in benign breast tissue and breast cancer tissues.

Studies have shown that G3BP1 is a cancer-promoting factor that is highly expressed in a variety of tumors and is associated with tumor progression (Xiong et al., 2019; Hu et al., 2021; Wang et al., 2021). We submitted G3BP1 to Xiantao web for analysis of its expression in pan-cancer. The results showed that G3BP1 expression was increased in most tumors (Figure 1A), and significantly higher in breast cancer than normal in 112 paired samples (Figure 1B). We then found that the RNA and protein expression levels of G3BP1 were significantly upregulated in primary breast cancer compared to normal individuals by the UALCAN database (Figure 1C). Furthermore, high expression of G3BP1 was associated with lower survival rates in breast cancer patients (Figures 1D,E). To verify the importance of G3BP1 in breast cancer, we examined the expression of G3BP1 in seven benign breast tissues and 68 breast invasive ductal carcinoma tissues by immunohistochemistry. Positive staining was detected in 53 tumor samples, but only in one benign breast tissue (Table 1), the representative images are shown in (Figure 1F). In addition, by analyzing the clinicopathological information we found that G3BP1 high expression was associated with metastasis of breast cancer patients (Table 2).

**TABLE 1:** Expression of G3BP1 in benign breast and breast cancer tissues.

	G3BP1			
	Total	Negative	Positive	*p* value
Benign Breast Tissue	7	6	1	0.002**
Breast Cancer Tissue	68	15	53	

IHC, staining results (+): positive, (-): negative. Statistically significant differences were indicated: **, *p*< 0.01.

The bold values in the table represent statistical significance

**TABLE 2:** Expression of G3BP1 in breast cancer tissues.

Parameters	G3BP1			
	Total	Negative	Positive	*p* value
Age				
<50	30	9	21	0.134
> = 50	38	6	32	
Tumor size (cm)				
<2	15	5	10	0.197
> = 2	53	10	43	
T stage				
1, 2	47	11	36	0.476
3, 4	21	4	17	
Metastasis				
Negative	33	11	22	0.029*
Positive	35	4	31	
c-erbB-2 protein				
Negative	44	7	37	0.090
Positive	24	8	16	
PCNA protein				
Negative	7	2	5	0.486
Positive	61	13	48	
p53 protein				
Negative	45	9	36	0.390
Positive	23	6	17	

IHC, staining results (+): positive, (-): negative. Statistically significant differences were indicated: *, *p*< 0.05.

The bold values in the table represent statistical significance

### G3BP1 regulates the proliferation and invasion of breast cancer cells

**FIGURE 2:**
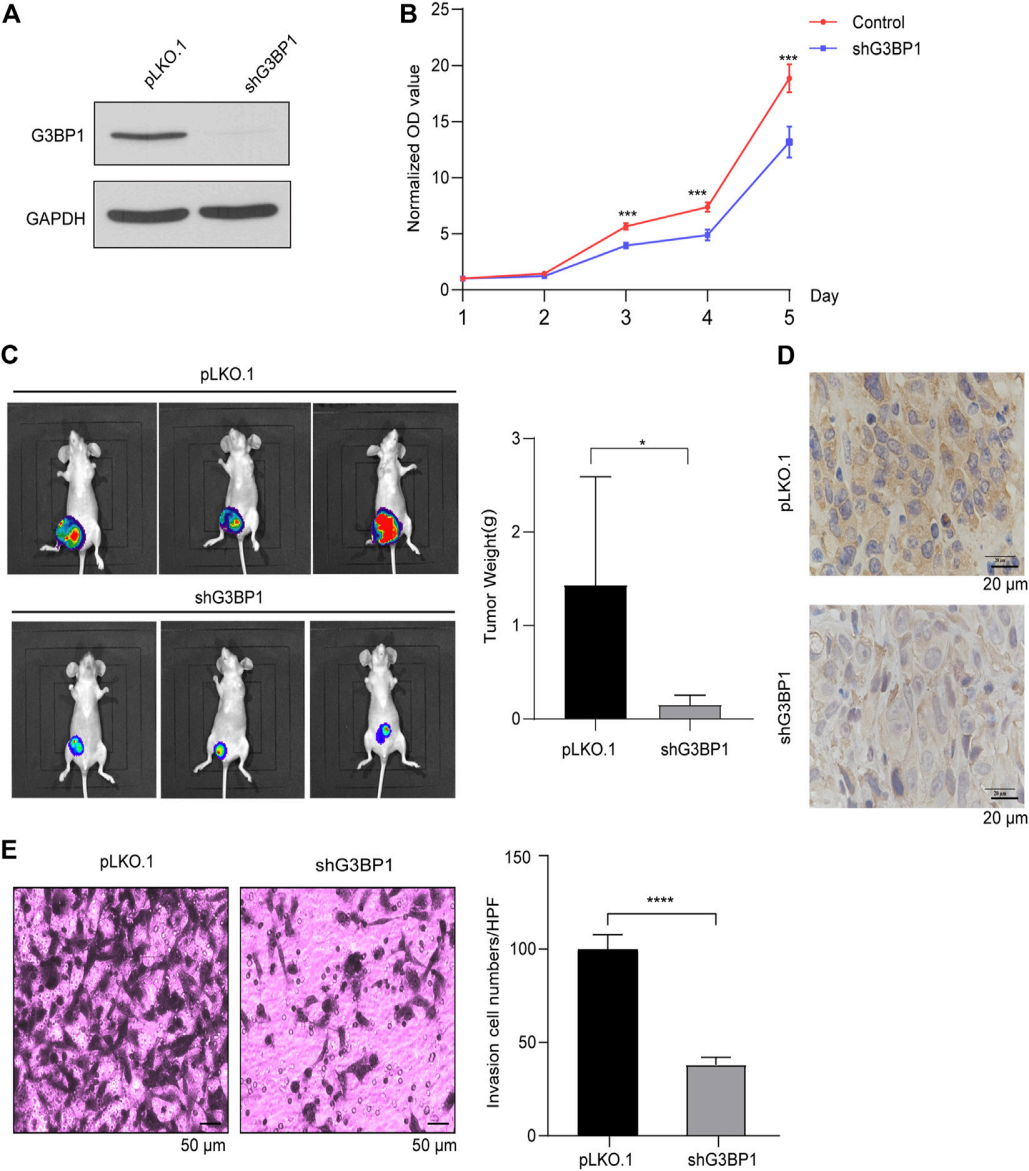
G3BP1 regulates the proliferation and invasion of breast cancer cells **(A)** Expression of G3BP1 in MDA-MB-231 stable knockdown cell line was analyzed by western blot [pLKO.1: control; shG3BP1: G3BP1 knockdown]. **(B)** Proliferation assay of control and G3BP1 knockdown group cells. Statistically significant differences were indicated: **, *p* < 0.01 by two-way ANOVA. **(C)** MDA-MB-231 cells transduced with luciferase-labeled G3BP1 knockdown (shG3BP1-luc) or control (pLKO.1-luc) were used to implant into the fourth pair of mammary fat pads of BALB/c nude mice for 10 weeks. The bioluminescence was detected by the IVIS imaging system [*n* = 5 per group]. Tumor growth was examined by the IVIS imaging system, and representative images are shown (left panel). The tumors of the two groups were exfoliated and weighed separately. Data were presented as mean ± SD, and statistically significant differences are indicated: *, *p* < 0.05 by Student’s *t*-test (right panel). **(D)** The expression of G3BP1 in tumor tissues of nude mice in both groups was stained by immunohistochemical staining. **(E)** Matrigel analysis of control and G3BP1 knockdown cells. Left panel: representative images of control and shG3BP1 cell invasion; Right panel: five regions were randomly selected to count the number of cells passing through the chamber in both groups. Data are expressed as mean ± SD by *t*-test. Statistically significant differences are indicated: ****, *p* < 0.0001.

We generated highly efficient lentiviral active particles using a lentiviral vector package of pLKO.1, infected MDA-MB-231 cells, and successfully established a G3BP1 stable knockdown cell line (Figure 2A). The proliferation ability of control and experimental cells was subsequently examined, and the results showed that the proliferation of breast cancer cells was significantly slowed down after G3BP1 was knocked down (Figure 2B), and this result was confirmed in a breast cancer xenograft model, where the tumor size and weight following G3BP1 knockdown were significantly reduced in nude mice (Figure 2C). We detected the expression of G3BP1 in nude mouse tumors by immunohistochemical staining and confirmed that the efficiency of G3BP1 down-expression was not restored during tumor growth (Figure 2D). Furthermore, the invasive ability of breast cancer cells was significantly inhibited by reducing the expression of G3BP1 (Figure 2E).

### G3BP1 is a key molecule that regulates the metastasis of breast cancer cells

**FIGURE 3:**
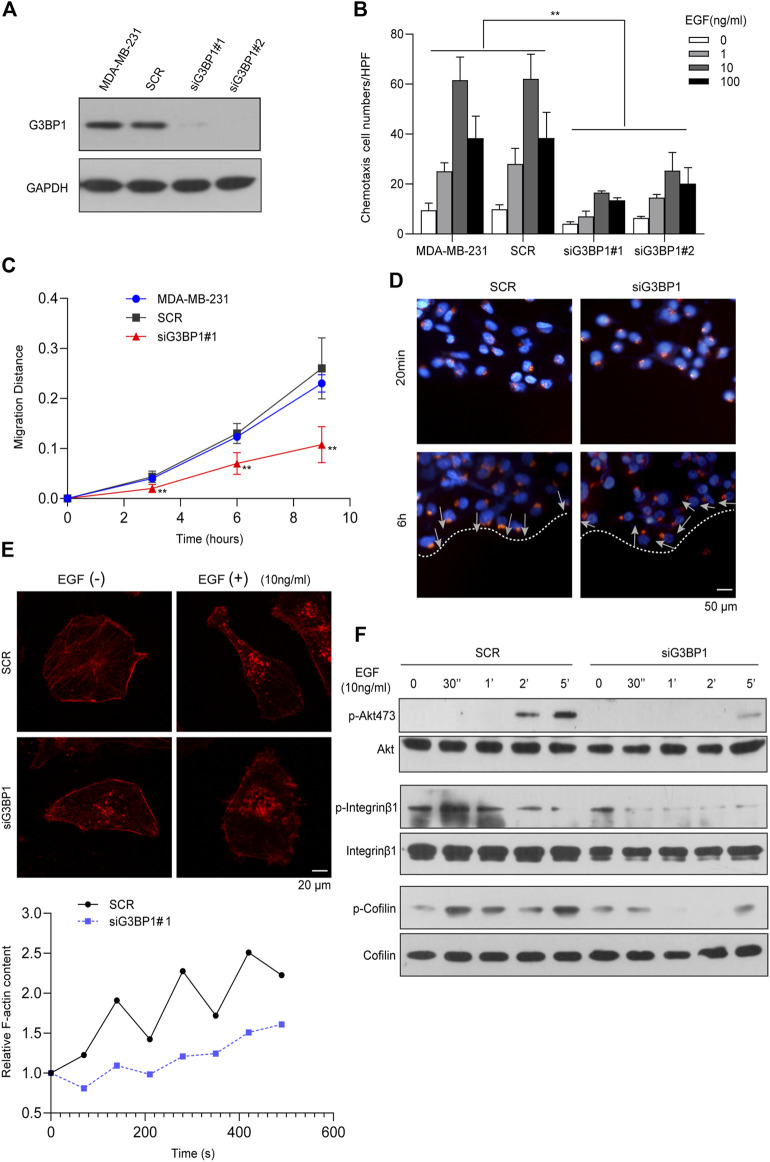
G3BP1 is a key molecule regulating metastasis in breast cancer cells **(A)** Western blot analysis of G3BP1 protein expression in cells transfected with siRNAs targeting G3BP1 (#1, #2) and promiscuous sequences (SCR). **(B)** EGF-induced chemotaxis of breast cancer cells was attenuated in siG3BP1 group. Statistically significant differences were indicated: **, *p* < 0.01. **(C)** Wound-healing assay of SCR and siG3BP1 cells. Statistically significant differences were indicated: **, *p* < 0.01 by two-way ANOVA. **(D)** Giantin immunofluorescence staining was used to detect Golgi polarity localization in the SCR and siG3BP1 groups. **(E)** Confocal microscopy was used to observe and record the aggregation of F-actin in the SCR and siG3BP1 groups. Upper panel: representative confocal microscopy images; Lower panel: biochemical staining for the detection of aggregated F-actin. **(F)** The effect of G3BP1 expression on Akt Ser473, Integrin β1 and Cofilin phosphorylation was analyzed by western blot.

We designed two independent siRNAs (#1, #2 siRNA) to target G3BP1 and used a scrambled sequence as a control (SCR). Transient transfection of both siRNAs specifically reduced G3BP1 expression in MDA-MB-231 cells, while transfection of the scrambled sequence did not (Figure 3A). Chemotaxis assays showed that reduced expression of G3BP1 significantly inhibited the epidermal growth factor (EGF)-induced chemotactic motility of breast cancer cells (Figure 3B). Next, we examined the cells in the control and siG3BP1 groups by scratch assay and recorded the change in scratch width at different times. The results showed that reducing the expression of G3BP1 could significantly inhibit the migratory ability of MDA-MB-231 cells (Figure 3C). The directional migration of cancer cells is closely related to the polarization status, and the migration of Golgi bodies contributes to the polarized migration of cells by promoting the effective transfer of Golgi-derived vesicles to the anterior part of the cells through microtubules (Vinogradova et al., 2009; Yadav and Linstedt, 2011). After scratching of the two groups of cells, they were fixed at 20 min and 6 h, then immunofluorescence staining was performed using Giantin (Golgi-specific molecular marker). We observed that the reduction of G3BP1 expression significantly inhibited the establishment of cell polarity in the directed movement of breast cancer cells (Figure 3D). Next, we treated both groups of cells with 10 ng/ml of EGF, stained F-actin with fluorescently labeled Phalloidin, and observed changes in intracellular actin polymerization by confocal microscopy. The results showed that the reduction of G3BP1 expression significantly inhibited EGF-induced actin polymerization in breast cancer cells (Figure 3E). Further, exploration of the role of G3BP1 in cellular signaling processes, showed the G3BP1 knockdown significantly inhibited the phosphorylation of Akt 473, integrin β1, and cofilin in EGF-induced breast cancer MDA-MB-231 cells as verified by western blot (Figure 3F).

### G3BP1 may regulate the movement of breast cancer cells by regulating PKCζ activation

**FIGURE 4:**
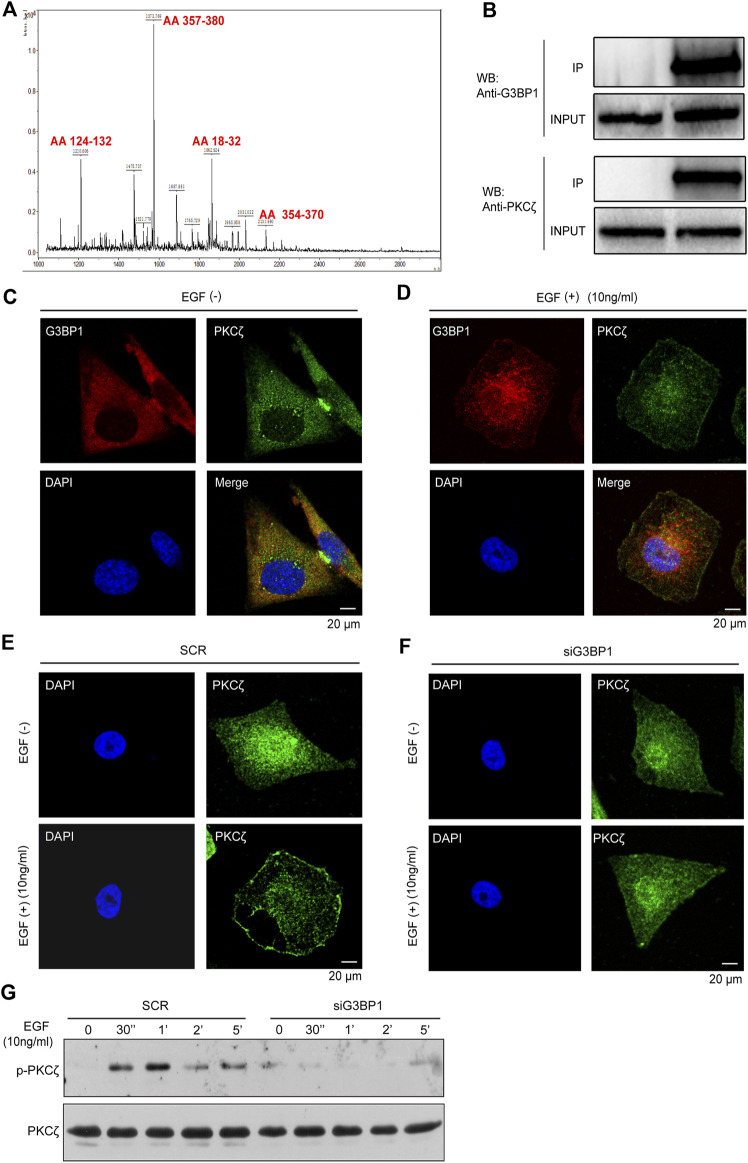
G3BP1 regulates the activation of PKCζ **(A)** Identification of G3BP1 as a PKCζ-interacting protein by immunoprecipitation and mass spectrometry. **(B)** MDA-MB-231 cell lysates were mixed with an anti-PKCζ antibody or IgG antibody. PKCζ and control immunoprecipitates were analyzed by western blot with anti-PKCζ and anti-G3BP1 antibodies. **(C)** Cellular localization of PKCζ and G3BP1 were assessed by confocal laser scanning microscopy. **(D)** Membrane translocation of G3BP1 and PKCζ in MDA-MB-231 cells after EGF stimulation was observed by confocal microscopy. **(E,F)** Effect of reduced expression of G3BP1 on PKCζ membrane translocation in MDA-MB-231 cells was analyzed by confocal microscopy. **(G)** The effect of G3BP1 expression on the phosphorylation of PKCζ in MDA-MB-231 cells was analyzed by western blot.

We first identified G3BP1 as a PKCζ-interacting protein by mass spectrometry (Figure 4A). Studies have shown that PKCζ plays an important role in tumors. We found that PKCζ expression was elevated in breast cancer, while there was a different expression pattern in other tumors (Supplementary Figure 1A). In addition, in 112 paired samples, the PKCζ expression levels were significantly higher in breast cancer than normal (Supplementary Figure 1B). Subsequently, the PKCζ was submitted to the UALCAN database to analyze its expression in unpaired samples, and the results showed that the mRNA and protein expression levels of PKCζ were significantly upregulated in primary breast cancer compared to normal (Supplementary Figure 1C). Previous studies have shown that PKCζ plays an important role in EGF-induced chemotaxis of cancer cells (Sun et al., 2005; Liu et al., 2009a). Therefore, we hypothesized that G3BP1 might regulate EGF-stimulated breast cancer cell motility through PKCζ. To confirm this hypothesis, we detected an interaction between G3BP1 and PKCζ using co-immunoprecipitation assay in breast cancer MDA-MB-231 cells (Figure 4B) and found the co-localization of G3BP1 and PKCζ in the cells by confocal microscopy (Figure 4C). Activation of PKCζ involves membrane translocation and phosphorylation. We used confocal microscopy to observe that G3BP1 and PKCζ co-translocated to the MDA-MB-231 cell membrane upon stimulation with 10 ng/ml EGF (Figure 4D). The distribution of PKCζ in siG3BP1 cells was unchanged after the reduction of G3BP1 with siRNA interference (Figure 4F). In contrast, PKCζ in SCR cells underwent significant membrane translocation in response to the same EGF stimulation (Figure 4E). These results suggested that the activation of PKCζ was hindered after reduction of G3BP1 expression. To further verify the effect of G3BP1 on PKCζ activation, phosphorylation of PKCζ in breast cancer cells was examined by western blot, which showed that knockdown of G3BP1 significantly impaired PKCζ phosphorylation (Figure 4G). These results demonstrate that G3BP1 interacts with PKCζ and regulates its activation.

## Discussion

Breast cancer is a common cancer in women and various biological factors affect the survival of breast cancer patients, including stage and tumor grade. With advances in treatment and improved screening techniques, early diagnosis and survival rates of breast cancer have been significantly improved. Although immunotherapy and targeted therapies are being actively developed, these therapies are effective in only a subset of patients. More seriously, among patients diagnosed with breast cancer at an early stage, the 5-year relative survival rate is close to 100%, but the 5-year survival rate for patients with metastatic breast cancer drops dramatically to 26% (Miller et al., 2019). Therefore, there is an unmet need to identify a molecular driver of tumor metastasis that holds promise as a novel therapeutic target for breast cancer.

Our previous research has demonstrated that YBX1 interacts with G3BP1 to upregulate its downstream protein SPP1 expression, thereby activating the NF-κB signaling pathway and thus promoting renal cell carcinoma (RCC) metastasis (Wang et al., 2019). The chronic inflammatory microenvironment in or around primary RCC sites contributes to the development of metastasis. We have previously reported that downregulation of G3BP1 can lead to disruption of IL-6-induced RCC migration and metastasis. Furthermore, *in vivo* orthotopic tumor xenografts results confirmed that downregulation of G3BP1 expression inhibited RCC tumor growth and metastasis in mice (Wang et al., 2018). In addition, G3BP1 has been reported to be highly expressed and play an important role in a variety of cancers. In ovarian cancer, the expression of G3BP1 is increased and loss of G3BP1 inhibits the proliferation, migration and invasion of ovarian cancer cells (Li et al., 2022). G3BP1 is significantly upregulated in colon cancer tissues and its high expression is closely associated with poor prognosis and clinical progression of colon cancer patients (Li et al., 2020). Studies have shown that G3BP1 knockdown inhibits glioblastoma-induced angiogenesis *in vivo* and promotes bortezomib-induced apoptosis by reducing stress granule formation in glioblastoma multiforme (Bittencourt et al., 2019). In addition, G3BP1 regulates the expression and secretion of senescent-associated secretory phenotype (SASP) by promoting cyclic GMP-AMP synthase (cGAS) to activate the NFκB and STAT3 pathways. G3BP1 deletion disrupts the cGAS pathway and blocks the expression of SASP factors, and these SASPless senescent cells disrupt senescence-mediated cancer cell growth *in vitro* and tumor growth *in vivo* (Omer et al., 2020). These results suggest that G3BP1 is involved in tumor progression through multiple pathways.

G3BP1 is highly expressed in various tumors and is associated with lymph node metastasis (Zhang et al., 2007). It has been reported to be interrelated with PTEN and involved in regulating cell proliferation and migration (Huang et al., 2005). In *Drosophila*, knockdown of the human G3BP1 homolog Rin resulted in a small eye polarity defect in compound eyes, a phenotype similar to that of RhoA mutant *Drosophila*, suggesting that G3BP1 may be a linkage between the Ras and Rho signaling pathways and involved in regulating the cytoskeleton (Pazman et al., 2000). Furthermore, high expression of G3BP1 correlates with high expression of Rhoc, which has an important role in cytoskeleton formation and reconstruction and is closely related to tumor metastasis (Schmitz et al., 2000; Cohen et al., 2003). In this study, we found that G3BP1 expression was upregulated in breast cancer. The knockdown of G3BP1 attenuated a series of metastasis-related properties of breast cancer cells, such as chemotaxis, migration, Golgi polarity localization, and actin polymerization, while the proliferation and invasive ability of breast cancer cells were also affected.

Protein kinase C (PKC), a member of the serine/threonine-protein kinase superfamily, is an important signal transduction molecule in cells and is involved in a variety of cellular functions, PKCζ is an atypical PKC (aPKC) consisting of four functional domains: protein domain, pseudosubstrate domain, cysteine-rich zinc finger domain, and kinase domain (Steinberg, 2008). Current studies indicate that PKCζ is important in EGF-induced breast cancer cell tropism. PKCζ can affect cytoskeletal rearrangement and play an important role in the process of cell polarization (Etienne-Manneville and Hall, 2003; Bertrand et al., 2010). In addition, it has been suggested that PKCζ may be involved in regulating the proliferation of melanoma (Ghosh et al., 2020). The expression of PTEN and G3BP1 is reciprocally influenced, PTEN is a downstream signaling molecule of EGFR that specifically dephosphorylates PIP3 (Bunney and Katan, 2010), while the activation of both PDK1 and AKT depends on PIP3 molecules on the cell membrane, interestingly, they are the key kinases for the activation of PKCζ (Wang et al., 2008; Liu et al., 2009b). In the present study, we found that G3BP1 and PKCζ interacted and co-localized in cells. Under EGF stimulation, G3BP1 and PKCζ co-translocated to the cell membrane, and knockdown of G3BP1 impaired both membrane translocation and phosphorylation of PKCζ. These results suggest that G3BP1 is an upstream regulatory molecule of PKCζ, but the exact mechanism still needs to be investigated in more depth.

In this study, we found that high expression of G3BP1 was associated with metastasis in breast cancer patients by immunohistochemical staining, and metastasis of tumors is the main cause of patient death. In addition, we found that G3BP1 expression was associated with survival in breast cancer patients using database, so G3BP1 may be a prognostic marker for breast cancer. The incidence of breast cancer ranks first among female malignant tumors, and metastasis often occurs at advanced stages of the disease, involving multiple organs and resulting in death. In this study, we found that knockdown of G3BP1 can inhibit the proliferation, invasion and metastasis of breast cancer cells, which suggests that G3BP1 may be a clinical therapeutic target.

## Conclusion

In this study, we found that knockdown of G3BP1 affected the proliferation and invasive capacity of breast cancer cells. Reduced expression of G3BP1 affected cellular properties associated with metastasis, including chemotaxis, polar localization, migration, and actin polymerization. Mechanistically, we identified G3BP1 as an interacting protein of PKCζ by mass spectrometry. G3BP1 interacted with PKCζ and promoted PKCζ membrane translocation and phosphorylation. In conclusion, G3BP1 played multiple functions in breast cancer cell proliferation and metastasis. Its activation of PKCζ may be a specific mechanism involved in this process.

## Data Availability

The original contributions presented in the study are included in the article/Supplementary Material, further inquiries can be directed to the corresponding authors.

## References

[B1] Abe J. Berk B. C. (2014). Novel mechanisms of endothelial mechanotransduction. Arterioscler. Thromb. Vasc. Biol. 34 (11), 2378–2386. 10.1161/ATVBAHA.114.303428 25301843 PMC4199910

[B2] An J. Peng C. Tang H. Liu X. Peng F. (2021). New advances in the research of resistance to neoadjuvant chemotherapy in breast cancer. Int. J. Mol. Sci. 22 (17), 9644. 10.3390/ijms22179644 34502549 PMC8431789

[B3] Bertrand F. Esquerre M. Petit A. E. Rodrigues M. Duchez S. Delon J. (2010). Activation of the ancestral polarity regulator protein kinase C zeta at the immunological synapse drives polarization of Th cell secretory machinery toward APCs. J. Immunol. 185 (5), 2887–2894. 10.4049/jimmunol.1000739 20679531

[B4] Bittencourt L. F. F. Negreiros-Lima G. L. Sousa L. P. Silva A. G. Souza I. B. S. Ribeiro Rima (2019). G3BP1 knockdown sensitizes U87 glioblastoma cell line to Bortezomib by inhibiting stress granules assembly and potentializing apoptosis. J. Neurooncol. 144 (3), 463–473. 10.1007/s11060-019-03252-6 31392596

[B5] Bos J. L. Rehmann H. Wittinghofer A. (2007). GEFs and GAPs: Critical elements in the control of small G proteins. Cell 129 (5), 865–877. 10.1016/j.cell.2007.05.018 17540168

[B6] Bray F. Laversanne M. Weiderpass E. Soerjomataram I. (2021). The ever-increasing importance of cancer as a leading cause of premature death worldwide. Cancer 127 (16), 3029–3030. 10.1002/cncr.33587 34086348

[B7] Bunney T. D. Katan M. (2010). Phosphoinositide signalling in cancer: Beyond PI3K and PTEN. Nat. Rev. Cancer 10 (5), 342–352. 10.1038/nrc2842 20414202

[B8] Chandrashekar D. S. Bashel B. Balasubramanya S. A. H. Creighton C. J. Ponce-Rodriguez I. Chakravarthi Bvsk (2017). Ualcan: A portal for facilitating tumor subgroup gene expression and survival analyses. Neoplasia 19 (8), 649–658. 10.1016/j.neo.2017.05.002 28732212 PMC5516091

[B9] Cohen M. Stutz F. Belgareh N. Haguenauer-Tsapis R. Dargemont C. (2003). Ubp3 requires a cofactor, Bre5, to specifically de-ubiquitinate the COPII protein, Sec23. Nat. Cell Biol. 5 (7), 661–667. 10.1038/ncb1003 12778054

[B10] Etienne-Manneville S. Hall A. (2003). Cell polarity: Par6, aPKC and cytoskeletal crosstalk. Curr. Opin. Cell Biol. 15 (1), 67–72. 10.1016/s0955-0674(02)00005-4 12517706

[B11] Gao T. Li M. Mu G. Hou T. Zhu W. G. Yang Y. (2019). PKCζ phosphorylates SIRT6 to mediate fatty acid β-oxidation in colon cancer cells. Neoplasia 21 (1), 61–73. 10.1016/j.neo.2018.11.008 30504065 PMC6277223

[B12] Ghosh S. Juin S. K. Nandi P. Majumdar S. B. Bose A. Baral R. (2020). PKCζ mediated anti-proliferative effect of C2 ceramide on neutralization of the tumor microenvironment and melanoma regression. Cancer Immunol. Immunother. 69 (4), 611–627. 10.1007/s00262-020-02492-0 31996991 PMC11027884

[B13] Guitard E. Parker F. Millon R. Abecassis J. Tocque B. (2001). G3BP is overexpressed in human tumors and promotes S phase entry. Cancer Lett. 162 (2), 213–221. 10.1016/s0304-3835(00)00638-8 11146228

[B14] Hartsink-Segers S. A. Beaudoin J. J. Luijendijk M. W. Exalto C. Pieters R. Den Boer M. L. (2015). PKCζ and PKMζ are overexpressed in TCF3-rearranged paediatric acute lymphoblastic leukaemia and are associated with increased thiopurine sensitivity. Leukemia 29 (2), 304–311. 10.1038/leu.2014.210 24990612 PMC4320296

[B15] Hu X. Xia K. Xiong H. Su T. (2021). G3BP1 may serve as a potential biomarker of proliferation, apoptosis, and prognosis in oral squamous cell carcinoma. J. Oral Pathol. Med. 50 (10), 995–1004. 10.1111/jop.13199 33987877

[B16] Huang Y. Wernyj R. P. Norton D. D. Precht P. Seminario M. C. Wange R. L. (2005). Modulation of specific protein expression levels by PTEN: Identification of AKAP121, DHFR, G3BP, Rap1, and RCC1 as potential targets of PTEN. Oncogene 24 (23), 3819–3829. 10.1038/sj.onc.1208527 15782128

[B17] Irvine K. Stirling R. Hume D. Kennedy D. (2004). Rasputin, more promiscuous than ever: A review of G3BP. Int. J. Dev. Biol. 48 (10), 1065–1077. 10.1387/ijdb.041893ki 15602692

[B18] Li M. Tang Y. Zuo X. Meng S. Yi P. (2022). Loss of Ras GTPase-activating protein SH3 domain-binding protein 1 (G3BP1) inhibits the progression of ovarian cancer in coordination with ubiquitin-specific protease 10 (USP10). Bioengineered 13 (1), 721–734. 10.1080/21655979.2021.2012624 34967276 PMC8805976

[B19] Li Y. Wang J. Zhong S. Li J. Du W. (2020). Overexpression of G3BP1 facilitates the progression of colon cancer by activating β‑catenin signaling. Mol. Med. Rep. 22 (5), 4403–4411. 10.3892/mmr.2020.11527 33000280 PMC7533501

[B20] Liu L. Z. Cheung S. C. Lan L. L. Ho S. K. Chan J. C. Tong P. C. (2010). The pivotal role of protein kinase C zeta (PKCzeta) in insulin- and AMP-activated protein kinase (AMPK)-mediated glucose uptake in muscle cells. Cell. Signal. 22 (10), 1513–1522. 10.1016/j.cellsig.2010.05.020 20570724

[B21] Liu Y. Wang B. Wang J. Wan W. Sun R. Zhao Y. (2009a). Down-regulation of PKCzeta expression inhibits chemotaxis signal transduction in human lung cancer cells. Lung Cancer 63 (2), 210–218. 10.1016/j.lungcan.2008.05.010 18701187

[B22] Liu Y. Wang J. Wu M. Wan W. Sun R. Yang D. (2009b). Down-regulation of 3-phosphoinositide-dependent protein kinase-1 levels inhibits migration and experimental metastasis of human breast cancer cells. Mol. Cancer Res. 7 (6), 944–954. 10.1158/1541-7786.MCR-08-0368 19531564

[B23] Ma L. Tao Y. Duran A. Llado V. Galvez A. Barger J. F. (2013). Control of nutrient stress-induced metabolic reprogramming by PKCζ in tumorigenesis. Cell 152 (3), 599–611. 10.1016/j.cell.2012.12.028 23374352 PMC3963830

[B24] Manna P. Jain S. K. (2013). PIP3 but not PIP2 increases GLUT4 surface expression and glucose metabolism mediated by AKT/PKCζ/λ phosphorylation in 3T3L1 adipocytes. Mol. Cell. Biochem. 381 (1-2), 291–299. 10.1007/s11010-013-1714-7 23749168 PMC4063451

[B25] Metcalfe K. A. Poll A. Royer R. Llacuachaqui M. Tulman A. Sun P. (2010). Screening for founder mutations in BRCA1 and BRCA2 in unselected Jewish women. J. Clin. Oncol. 28 (3), 387–391. 10.1200/JCO.2009.25.0712 20008623

[B26] Miller K. D. Nogueira L. Mariotto A. B. Rowland J. H. Yabroff K. R. Alfano C. M. (2019). Cancer treatment and survivorship statistics, 2019. Ca. Cancer J. Clin. 69 (5), 363–385. 10.3322/caac.21565 31184787

[B27] Omer A. Barrera M. C. Moran J. L. Lian X. J. Di Marco S. Beausejour C. (2020). G3BP1 controls the senescence-associated secretome and its impact on cancer progression. Nat. Commun. 11 (1), 4979. 10.1038/s41467-020-18734-9 33020468 PMC7536198

[B28] Oubaha M. Lin M. I. Margaron Y. Filion D. Price E. N. Zon L. I. (2012). Formation of a PKCζ/β-catenin complex in endothelial cells promotes angiopoietin-1-induced collective directional migration and angiogenic sprouting. Blood 120 (16), 3371–3381. 10.1182/blood-2012-03-419721 22936663 PMC3476545

[B29] Pazman C. Mayes C. A. Fanto M. Haynes S. R. Mlodzik M. (2000). Rasputin, the Drosophila homologue of the RasGAP SH3 binding protein, functions in ras- and Rho-mediated signaling. Development 127 (8), 1715–1725. 10.1242/dev.127.8.1715 10725247

[B31] Rajalingam K. Schreck R. Rapp U. R. Albert S. (2007). Ras oncogenes and their downstream targets. Biochim. Biophys. Acta 1773 (8), 1177–1195. 10.1016/j.bbamcr.2007.01.012 17428555

[B32] Schmitz A. A. Govek E. E. Bottner B. Van Aelst L. (2000). Rho GTPases: Signaling, migration, and invasion. Exp. Cell Res. 261 (1), 1–12. 10.1006/excr.2000.5049 11082269

[B33] Steinberg S. F. (2008). Structural basis of protein kinase C isoform function. Physiol. Rev. 88 (4), 1341–1378. 10.1152/physrev.00034.2007 18923184 PMC2899688

[B34] Sun R. Gao P. Chen L. Ma D. Wang J. Oppenheim J. J. (2005). Protein kinase C zeta is required for epidermal growth factor-induced chemotaxis of human breast cancer cells. Cancer Res. 65 (4), 1433–1441. 10.1158/0008-5472.CAN-04-1163 15735031

[B35] Sung H. Ferlay J. Siegel R. L. Laversanne M. Soerjomataram I. Jemal A. (2021). Global cancer statistics 2020: GLOBOCAN estimates of incidence and mortality worldwide for 36 cancers in 185 countries. Ca. Cancer J. Clin. 71 (3), 209–249. 10.3322/caac.21660 33538338

[B36] Vinogradova T. Miller P. M. Kaverina I. (2009). Microtubule network asymmetry in motile cells: Role of golgi-derived array. Cell Cycle 8 (14), 2168–2174. 10.4161/cc.8.14.9074 19556895 PMC3163838

[B37] Wang C. Cui Q. Du R. Liu S. Tian S. Huang H. (2021). Expression of G3BP1 in benign and malignant human prostate tissues. Transl. Androl. Urol. 10 (4), 1665–1675. 10.21037/tau-20-1450 33968655 PMC8100838

[B38] Wang J. Wan W. Sun R. Liu Y. Sun X. Ma D. (2008). Reduction of Akt2 expression inhibits chemotaxis signal transduction in human breast cancer cells. Cell. Signal. 20 (6), 1025–1034. 10.1016/j.cellsig.2007.12.023 18353613

[B39] Wang Y. Fu D. Chen Y. Su J. Wang Y. Li X. (2018). G3BP1 promotes tumor progression and metastasis through IL-6/G3BP1/STAT3 signaling axis in renal cell carcinomas. Cell Death Dis. 9 (5), 501. 10.1038/s41419-018-0504-2 29717134 PMC5931548

[B40] Wang Y. Su J. Wang Y. Fu D. Ideozu J. E. Geng H. (2019). The interaction of YBX1 with G3BP1 promotes renal cell carcinoma cell metastasis via YBX1/G3BP1-SPP1- NF-κB signaling axis. J. Exp. Clin. Cancer Res. 38 (1), 386. 10.1186/s13046-019-1347-0 31481087 PMC6720408

[B41] Xiong R. Gao J. L. Yin T. (2019). G3BP1 activates the TGF-β/Smad signaling pathway to promote gastric cancer. Onco. Targets. Ther. 12, 7149–7156. 10.2147/OTT.S213728 31564899 PMC6730608

[B42] Yadav S. Linstedt A. D. (2011). Golgi positioning. Cold Spring Harb. Perspect. Biol. 3 (5), a005322. 10.1101/cshperspect.a005322 21504874 PMC3101843

[B43] Zang G. Mu Y. Gao L. Bergh A. Landstrom M. (2019). PKCζ facilitates lymphatic metastatic spread of prostate cancer cells in a mice xenograft model. Oncogene 38 (22), 4215–4231. 10.1038/s41388-019-0722-9 30705401 PMC6756056

[B44] Zhang H. Z. Liu J. G. Wei Y. P. Wu C. Cao Y. K. Wang M. (2007). Expression of G3BP and RhoC in esophageal squamous carcinoma and their effect on prognosis. World J. Gastroenterol. 13 (30), 4126–4130. 10.3748/wjg.v13.i30.4126 17696235 PMC4205318

