# Supplementary figures and images for: G3BP1 regulates breast cancer cell proliferation and metastasis by modulating PKCζ
Source: Front Genet. 2022 Oct 18;13:1034889. doi: 10.3389/fgene.2022.1034889 (PMC9623284; doi:10.3389/fgene.2022.1034889)

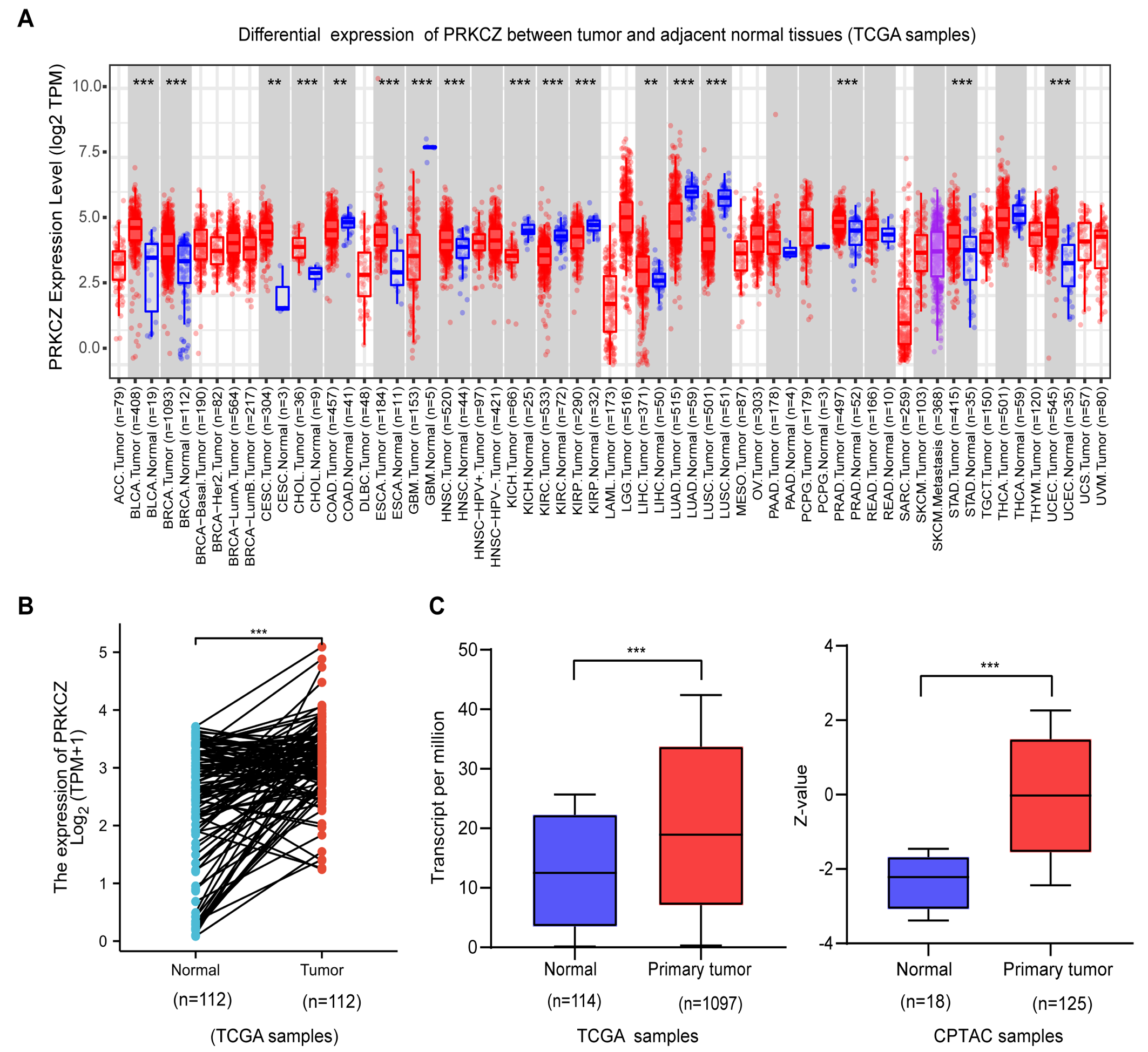

Supplement: Supplementary file 1 [file Image1.TIF]
