# Supplementary material for: G3BP1 regulates breast cancer cell proliferation and metastasis by modulating PKCζ
Source: Front Genet. 2022 Oct 18;13:1034889. doi: 10.3389/fgene.2022.1034889 (PMC9623284; doi:10.3389/fgene.2022.1034889)
Supplement: Supplementary file 2 [file DataSheet1.docx]

Supplementary Material

**Summary**

The primer sequences of pLKO.1-shG3BP1 and siG3BP1 and Supplemental Figure 1.

**Supplemental Table 1.** The primer sequences of pLKO.1-shG3BP1

|  | Primers sequences (5′ to 3′) |
| --- | --- |
|  | F:CCGGCGGGAATTTGTGAGACAGTATCGGGATCCAATACTGTCTCACAAATTCCCGTTTTTTG |
|  | R:AATTCAAAAAACGGGAATTTGTGAGACAGTAGGGTTATCCCGAGACTGTCTCACAAATTCCCG |

**Supplemental Table 2.** The primer sequences of siG3BP1

|  | Primers sequences (5′ to 3′) |
| --- | --- |
|  | #1 F: CGGGAAUUUGUGAGACAGUAUtt |
|  | R: ttAUACUGUCUCACAAAUUCCCG  #2 F: CCACCUCAUGUUGUUAAAGUAtt  R: ttUACUUUAACAACAUGAGGUGG |


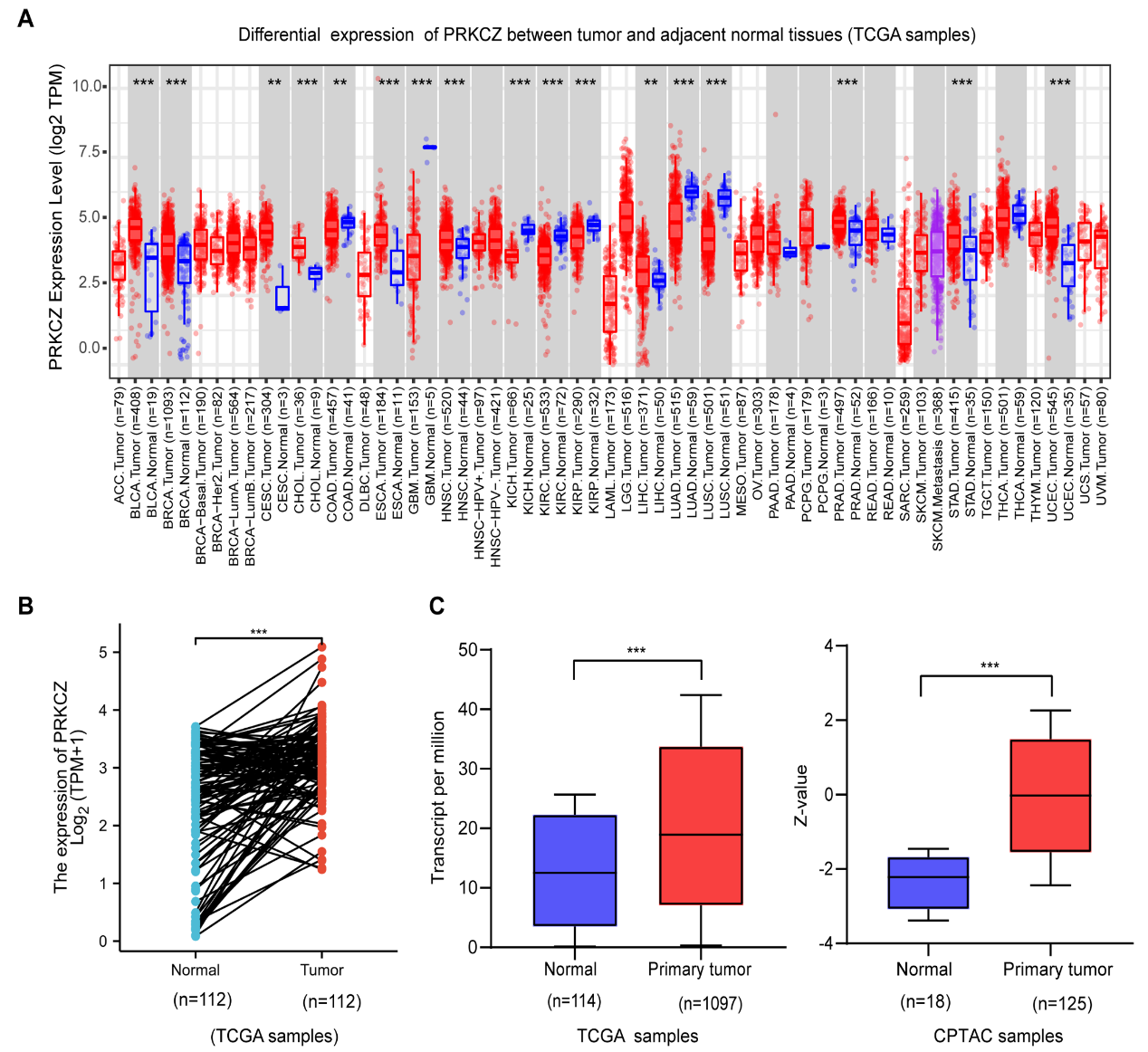


**Supplemental Figure 1 PKCζ is highly expressed in breast cancer** (**A**) Expression levels of PKCζ mRNA in pan-cancer (https://cistrome.shinyapps.io/timer/) **, *p*<0.01, ***, *p*<0.001. (**B**) Expression of PKCζ mRNA in 112 groups of breast cancer and normal paired samples (https://www.xiantao.love) ***, *p*<0.001. (**C**) The mRNA and protein expression of PKCζ between normal and primary breast cancer (http://ualcan.path.uab. edu) ***, *p*<0.001, ***, *p*<0.001.
